# 3D Virtual modelling, 3D printing and extended reality for planning of implant procedure of short-term and long-term mechanical circulatory support devices and heart transplantation

**DOI:** 10.3389/fcvm.2023.1191705

**Published:** 2023-07-28

**Authors:** Alexander Stepanenko, Laura Maroto Perez, Jordi Candela Ferre, Cristina Ybarra Falcón, Enrique Pérez de la Sota, Jose Alberto San Roman, Alfredo Redondo Diéguez, Carlos Baladron

**Affiliations:** ^1^ Cardiology Department, Hospital Clínico Universitario de Valladolid, Valladolid, Spain; ^2^ Centro de Investigación Biomédica en Red de Enfermedades Cardiovasculares (CIBERCV), Madrid, Spain; ^3^ Cardiovascular Surgery, Hospital Recoletas Campo Grande, Valladolid, Spain; ^4^ Cardiovascular Surgery, Hospital 12 de Octubre, Madrid, Spain; ^5^ Vall3DLab, Hospital Clínico Universitario de Valladolid, Valladolid, Spain; ^6^ Cardiology Department, University Hospital of Santiago, Santiago de Compostela, Spain

**Keywords:** 3D printing, virtual device implant, ventricular assist device, heart transplantation, virtual reality, augmented reality

## Abstract

**Introduction:**

The use of three-dimensional (3D) reconstruction and printing technology, together with extended reality applied to advanced heart failure adult patients with complex anatomy, is rapidly spreading in clinical practice. We report practical experience with application to acute and chronic heart failure: planning and performing mechanical circulatory device insertion or heart transplantation.

**Methods:**

From November 2019 until February 2022, 53 3D virtual biomodels were produced for intervention planning (using Virtual/Augmented Reality and/or 3D printing), following a specific segmentation and preprocessing workflow for biomodelling, in patients with advanced heart failure due to structural heart disease or cardiomyopathies. Four of those patients were complex cases requiring mechanical circulatory support implant procedures in our center.

**Results:**

One short-term and three long-term ventricular assist device system were successfully clinically implanted after application of this technique. In other two cases with extremely high procedural risk, visualized after application of this multimodality imaging, heart transplantation was elected.

**Conclusion:**

3D printing based planning and virtual procedure simulation, are of great importance to select appropriate candidates for mechanical circulatory support in case of complex patient anatomy and may help to diminish periprocedural complications. Extended reality represents a perspective tool in planification of complex surgical procedures or ventricular assist device insertion in this setting.

## Introduction

Along the past two decades, the application of three-dimensional (3D) printing technology has become an extended tool for the physicians. Initially, patient specific physical models had been shown to be a useful tool in maxillofacial surgery, reconstructive surgery, orthopedics, and in pediatric cardiac operations (1, 2). By using this technology, surgical templates or customized implants can be tested on the models, or complex anatomic structures can be revised before the intervention. More recently, this approach has also been adopted for complex adult cardiac surgery cases (3–5). There is evidence that 3D printed biomodels accurately replicate anatomy and that planning over physically printed models can effectively change and improve the surgical approach in near the 50% of the patients in specific and challenging cases (6, 7).

As the amount of adult population with congenital heart failure is continuously increasing, the onset of advanced heart failure in their 3–4 decade of life is common. Multimodality imaging for this population with complex anatomy to plan heart transplantation or Ventricular Assist Device (VAD) implantation becomes widely implemented (8).

Lately, the application of 3D printing in the field of adult advanced heart failure has rapidly evolved from anecdotal cases to a routine tool for planification and simulation of both surgical and interventional procedures (9).

3D printing is still a relatively time- and labor -intensive process. Therefore, use of created 3D virtual model allows virtually testing of device fitting in patient's chest and proper inflow orientation toward to atrioventricular valve. The created patient-specific models provide valuable spatial information regarding the anatomy as whole, including endocardial calcifications, relation to major structures as intraventricular septum, coronary arteries, orientation to heart valves and extra cardiac device interaction to the chest wall and diaphragm.

The aim of this review is highlighting the role of 3D printing, virtual intervention and use of extended reality for periprocedural planning in adults with advanced heart failure requiring short-term or long-term mechanical circulatory support or heart transplantation.

Between the years 2019 and 2022, we produced and printed 53 three-dimensional biomodels (Vall3DLab, Valladolid, Spain). Source data was preoperative computed tomography imaging, acquired in adult patients with advanced heart failure due to structural heart disease or cardiomyopathies scheduled for surgical or percutaneous intervention. These models were produced following a specific segmentation and preprocessing workflow designed for accurate three-dimensional analysis, combining semi-automatic algorithms and manual procedures. This work is focused on four of these patients that were evaluated for VAD implantation in our center.

## Three-dimensional printing workflow

**Figure 1:**
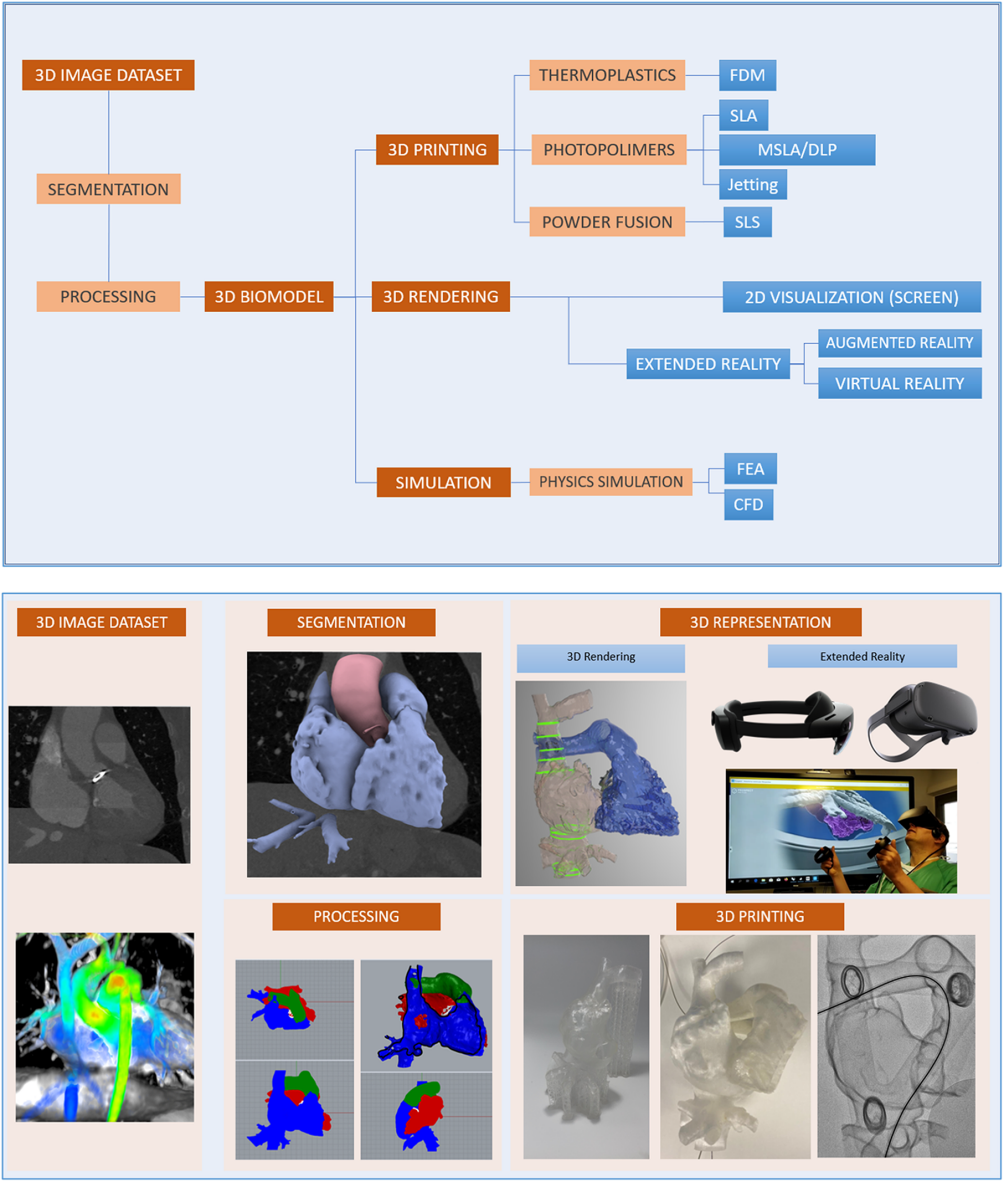
Three-dimensional printing and virtual model creation.

**Figure 2:**
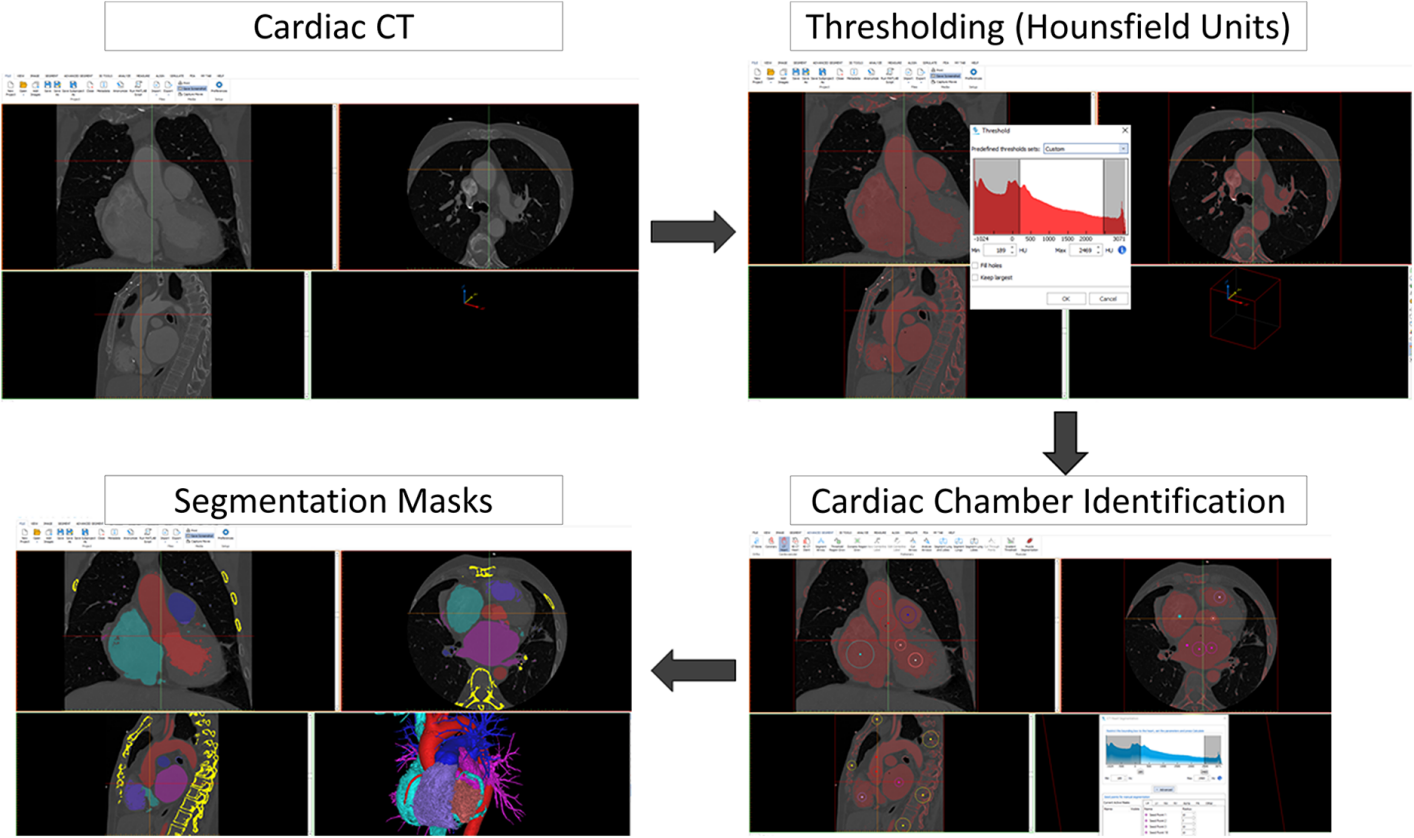
Segmentation with the Mimics Innovation Suite using the cardiac segmentation module.

**Figure 3:**
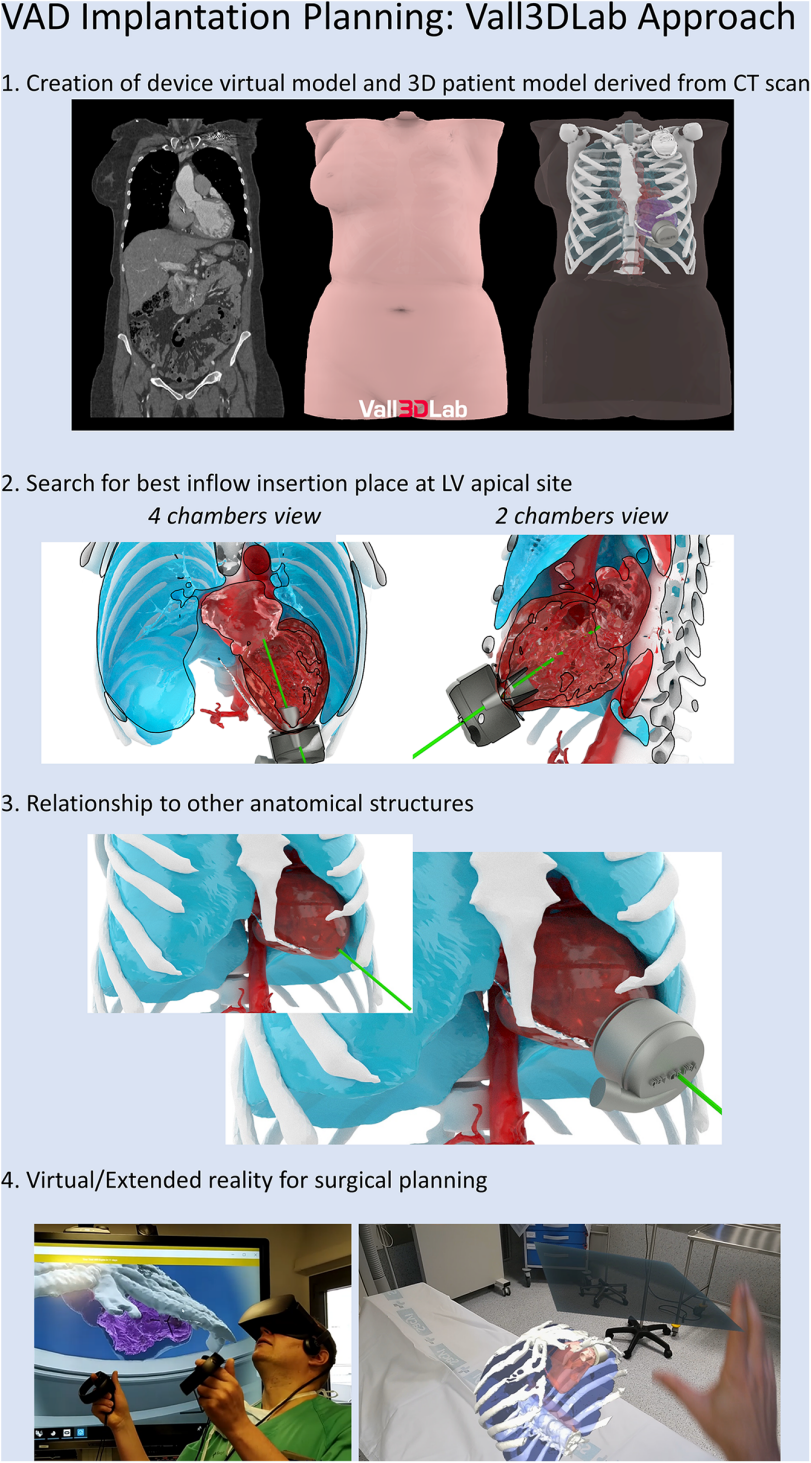
Central illustration. Planification for virtual VAD implantation.

The elaboration of heart 3D printed-phantoms comprises several steps (Figure 1):

1. Volumetric image data acquisition and segmentation
2. 3D postprocessing and modelling to the final 3D image renders
3. 3D printing
4. Use of augmented or extended reality
1. **Volumetric image data acquisition and segmentation: Sources of image for three-dimensional modeling**:
  Biomodels can be extracted from a variety of medical imaging modalities although computed tomography (CT) and magnetic resonance imaging (MRI) are the most commonly used due to high Signal-to-Noise Ratio (SNR) and spatial resolution. In volumetric medical images, the voxel is the minimum unit (equivalent to the pixel in two-dimensional digital images). Each voxel is assigned a signal intensity that represents the average value of the tissue in terms of Hounsfield units (CT) or signal intensity (MRI) and commonly represented as a gray scale. By means of co-registration techniques, it is even possible to combine different medical images to generate a biomodel by taking advantage of the strengths of each technique.
  The source data for all models presented in this work is CT. Acquisition was performed with a General Electric LightSpeed VCT (25 cm Field of view, 100–120 kV, 600 mA–700 mA, 0.35s rotation time, 0.625 mm slice thickness, 512 × 512 matrix). Raw DICOM is employed for exporting data. No specific acquisition protocol is employed for model generation: imaging is performed according to standard clinical procedures, and then a quality check procedure is carried out by the 3D operator in order to guarantee that data is appropriate and adherent to the guidelines for medical 3D printing of the Radiological Society of North America (RSNA) (10).
  The process of extracting an anatomic structure of interest from the volumetric image is known as segmentation. Computer programs for segmentation allow the delimitation of structures of interest from adjacent ones. They are based on two strategies for making this differentiation: intensity-based segmentation and shape-based segmentation. For example, in aortic angiography, intravascular light can be identified by taking advantage of the large difference in Hounsfield units of the contrast compared to adjacent structures. Depending on the structures to be segmented, it will be necessary for the CT acquisition to be performed at a certain contrast phase. On the other hand, the principle behind shape-based segmentation is that the general geometry of an organ is known. This allows the software to recognize and separate structures based on an analysis of its morphology.
  Several software options are available in the market: from the open source option SLICER (www.slicer.org) to commercial applications approved by the Federal Drug Administration (FDA) for medical use, such as Mimics Innovation Suite Medical (Materialise, Leuven, Belgium). The latter has been used for this work.
  In Figure 2, an example of segmentation with the Mimics Innovation Suite using the cardiac segmentation module is shown. First, a Hounsfield unit threshold is defined; in this case, the selected area includes the intravascular light, but also bone tissue. In the next step, the cardiac segmentation module a set of marks manually positioned inside the structures of interest (known as seeds) as the starting point for an algorithm that intelligently generates a first version of those structures(in this case, for example, discarding bone tissue and keeping muscle and vessels). The model is finalized manually: the operator adds and removes specific sections of the images that could have been missed by the semi-automatic algorithms. The segmented model is then exported to a Standard Tessellation Language (STL) file to continue with model production. When procedure planning requires hollow heart chambers, tissue and contrasted blood are segmented separately, and then the latter subtracted from the former to maintain myocardium and chamber dimensions.
  Experts of the ISO9001-certified ICICORELAB corelab (www.icicorelab.es) performed image segmentation for this work. They are cardiologists with a specialization in cardiac imaging and a track record of participation in state-of-the-art image analysis tasks both for clinical and research purposes. Inside the ICICORELAB, inter- and intra-operator variability analysis are routinely performed for a variety of techniques (although they have not been performed yet for biomodel production).
2. **Three-dimensional post processing and modelling**: The 3D mesh of the segmented structure is further processed using a computer assisted design (CAD) software (Rhinoceros 3D 7, Robert McNeel and Associates for Windows, Washington DC, USA) to create a printable 3D-phantom. This step includes (1) fixing and smoothing the mesh (reducing clutter and noise); (2) hollowing vessels in models which do not include heart chambers (this is done via 1 mm-width extrusion in order to keep internal dimensions); (3) adding holes (or slicing the model in several parts) to facilitate visual inspection, manipulation and printing of closed structures.
  In order to reduce mesh size and computational requirements while maintaining accuracy of the 3D model, adaptive mesh topology optimization tools available in Rhinoceros 7 (Mcneel & Associates) were applied. The final post-processed 3D model was imported into the segmentation software, and the model accuracy was checked by overimposing the outline of the model over the originally acquired medical image.
3. **Three-dimensional printing**: 3D printing (also known as additive manufacturing) is the process of creating the object by adding layer-upon-layer of material. Currently, there is a variety of different 3D printing technologies with different strengths and weaknesses. Initially, we used the most affordable and widely adopted technologies, the Fused Deposition Modeling (FDM) and Stereolitography (SLA); in the former, a plastic filament is fused through a hot extruder and in the latter an ultraviolet-curable resin is solidified under an appropriate light source -normally a laser diode-). Other technologies, such as Selective Laser Sintering or Jetting printing are high-end technologies far more expensive and were not used.
  For most of produced the models (and the four cases detailed in this work), we have employed SLA, a highly accurate yet affordable technology, with a Formlabs Form 3 printer (Formlabs, Massachusetts, USA), combining rigid (Clear V4, White V4) and flexible (Flexible 80A, Elastic 50A) resins. The selected materials to print the 3D model depend on the specific purpose of the model, creating multimaterial 3D phantoms according to the proprieties of the different tissues that compound the model, to mimic the strain stress behavior of certain tissues (11). This is only used as an approximate reference of tissue properties (e.g.: bone vs. vessels) and is in no way intended for accurate flexibility or deformation analysis.
  A 3D printing management software is employed in order to configure printing parameters (e.g.: layer thickness), adding holes for resin drainage and supports for overhangs. Formlabs PreForm software was employed for this task. Printing configuration varied from model to model depending on the specific conditions and purpose, but in all cases, the RSNA guidelines (10) were followed.
4. **Use of augmented or extended reality (Figure 3):** Another innovative aspect in the visualization of medical images is known as augmented reality, a term that encompasses different technologies: virtual reality, augmented reality, and mixed reality. Virtual and augmented reality headsets were employed for this end. Virtual reality generates a whole synthetic and interactive 3D environment where the operator can examine the biomodel, and interact with it with its own hands as if it was a real object. In augmented reality (Microsoft Hololens glasses), the 3D synthetic biomodels are “holographicaly” overlayed over the real world, so they can also interact with other elements of the operation room or cath lab.
  The application of mixed reality is gaining momentum in the field of surgery and interventional procedures, taking advantage of the spatial location capability. It allows positioning biomodels in real-time referenced in the real anatomical position of the patient (see Supplementary Video S1).

3D Biomodel inspection could be performed on a conventional 3D workstation operated on a traditional 2D screen. However, AR/VR and 3D printed models improve perception and provide a much more natural interaction with the model. This improved perception results in a better evaluation of the patient. In the results section, we will show how the selected surgical approach is changed after 3D biomodel examination by the operator.

## Right ventricular cavity reconstruction

**Figure 4:**
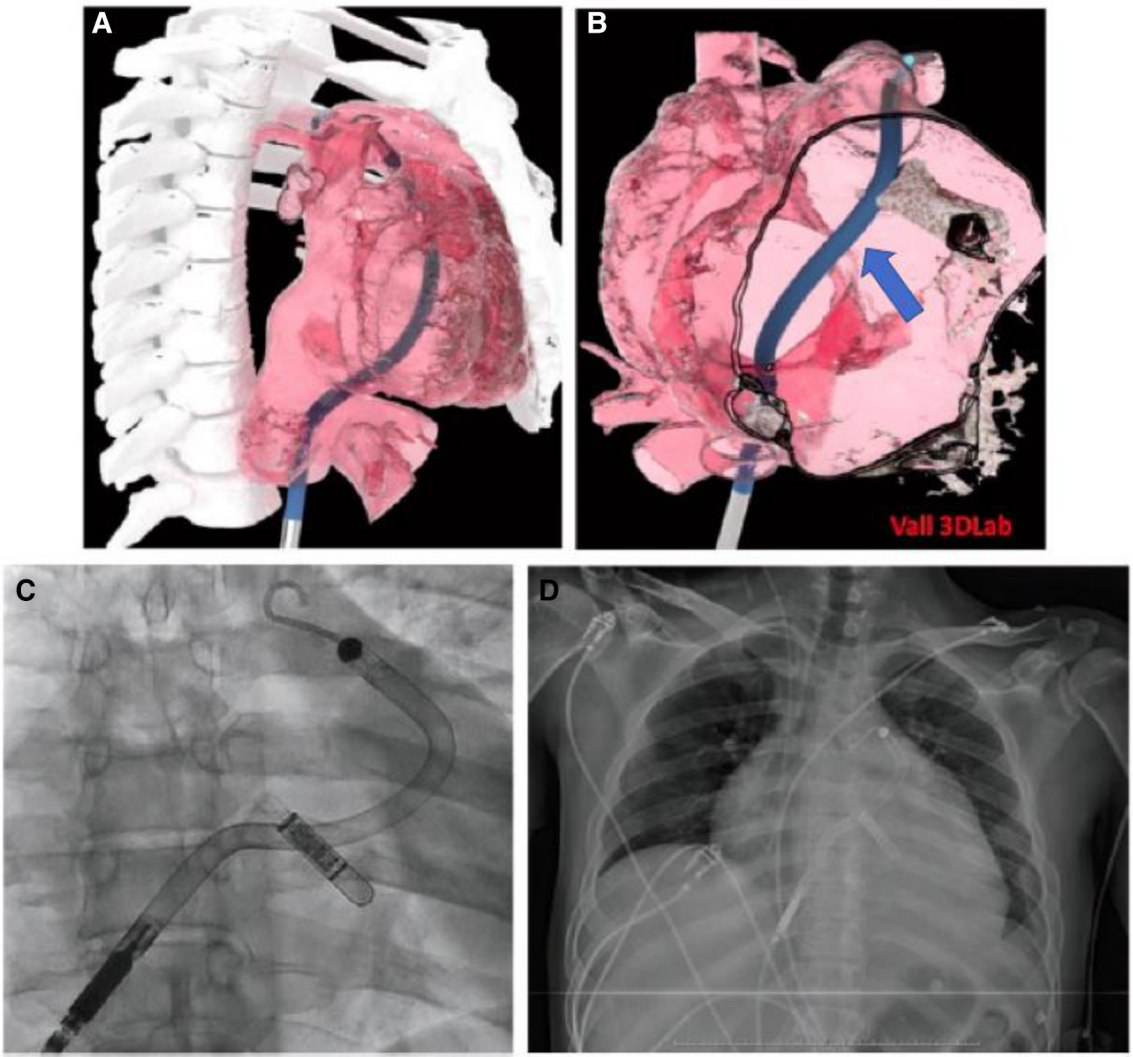
(**A,B**) 3-D reconstruction of the right sided chambers. Suitably of the right ventricle Impella RP positioning and adequate outflow location at pulmonary artery trunk. Blue arrow pointing at middle part of device passing through tricuspid valve. (**C,D**) Position of the Impella RP inlet within the dilated right atrium and the outlet within the main pulmonary artery (LINQ device observed).

**Case 1**: A male patient with an Ebstein type C anomaly who suffered end-stage heart failure was listed for heart transplantation at our institution. As his clinical status worsened and he became inotrope-dependent (INTERMACS 3 -Interagency Registry for Mechanically Assisted Circulatory Support 3-), the Heart Team discussed the use of multimodality imaging to prove the suitability of a percutaneous short-term circulatory support device (Impella RP, Abiomed, Intl.). A 3D biomodel of right ventricular cavity, pulmonary artery (PA), right atrium and inferior vena cava was segmented from the end-diastolic phase of an electrocardiographically gated CT scan using software Mimics innovation 23 (Materialise, Leuven, Belgium). Further, processing and modeling of Impella RP device was performed with Rhinoceros 3D 7. A virtual Impella RP device was virtually implanted with outflow orifice setted above pulmonary valve. It was possible perform a virtual implantation of the device, with location of the outflow 27 mm above the pulmonary valve, in the PA trunk. Based on the competence of pulmonary valve and suitability in the 3D reconstruction of the right ventricle (Figures 4A,B), we decided to proceed with Impella RP implantation. Regardless of the challenging anatomy, the device was correctly and uneventfully fluoroscopic-guided deployed, so as to position the inlet within the dilated right atrium and the outlet within the main pulmonary artery (Figures 4C,D) (12). After extended support duration (16 days as compassionate use out of its certification) and bedside explant, a mechanical unloading induced a partial recovery that allowed him to be transplanted 4 months later electively (at clinical status INTERMACS 5 -Interagency Registry for Mechanically Assisted Circulatory Support 5) (13).

The main objective of 3D modelling was to resolve obstacles of Impella RP positioning. Right Ventricle dilatation may difficult stable device outflow position at pulmonary artery main trunk and cause therefore procedure failure. Hence, virtual implant helped to gain important skills during virtual device positioning and prepare team for the implant (election of support wire, bail-out plans, surgical back-up).

## Left ventricle cavity reconstruction

**Figure 5:**
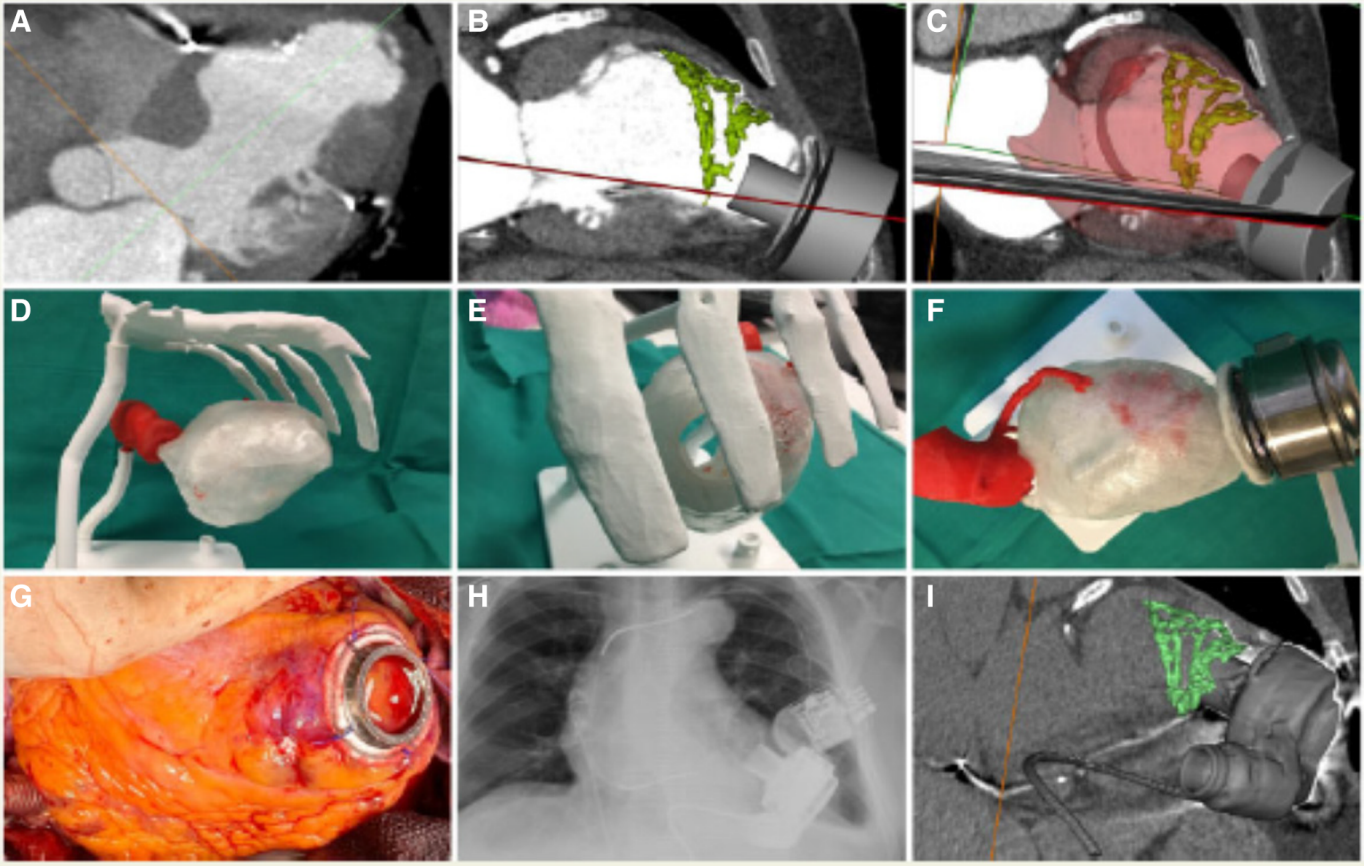
(**A–C**) HeartMateIII fitting patient's chest 3D-reconstruction. (**D–F**) HMIII inflow implanted in 3D printed specific-patient phantom. (**G–I**) Final HMIII position in patient's heart.

**Case 2**: A 73-year-old male with an atypically remodelled left ventricle cavity, a calcified septo-apical aneurysm and an apical aneurism was admitted as candidate for long term left ventricular assist device (LVAD) implantation. A 3D model of Heartmate 3^™^ (Abbott, Intl) intrapericardially placed pump was designed and virtually implanted in an optimal position in a 3D biomodel of the patient's heart: with the inflow cannula avoiding the endocardial calcification and pointing towards the mitral valve (Figure 5A). The relation of the HeartMate 3^™^ with the chest wall predicted the need of creation of the extrapericardial pump pocket. At the same time, extended reality was used to enhance surgical view. During surgery, apical ring was first sewed and then, myocardium cored at planned point of the apical aneurysm. The pump body was accommodated extrapericardially, resulting in optimal position of the inflow cannula toward to mitral valve after chest closure (Figures 5B,C). The LVAD flow ranged between 4 and 4.5 L/min in the absence of relevant suction events. The 3D biomodels and 3D printed patient-specific phantoms, as well as extended reality, were useful to provide optimal pre-surgical planning (Figures 5D–F):

1. Avoidance of endocardial calcium by choosing an atypical inflow insertion point: laterally, as usual, but preserving optimal orientation of the inflow cannula to atrioventricular valve.
2. The need for creation of an extrapericardial pocket to accommodate the position of the intrapericardially designed device.

The application of this multimodality imaging biomodel contributed to the successful implantation of the modern LVAD (14). After rehabilitation, the patient was discharged home and is ongoing for over 3 years.

**Figure 6:**
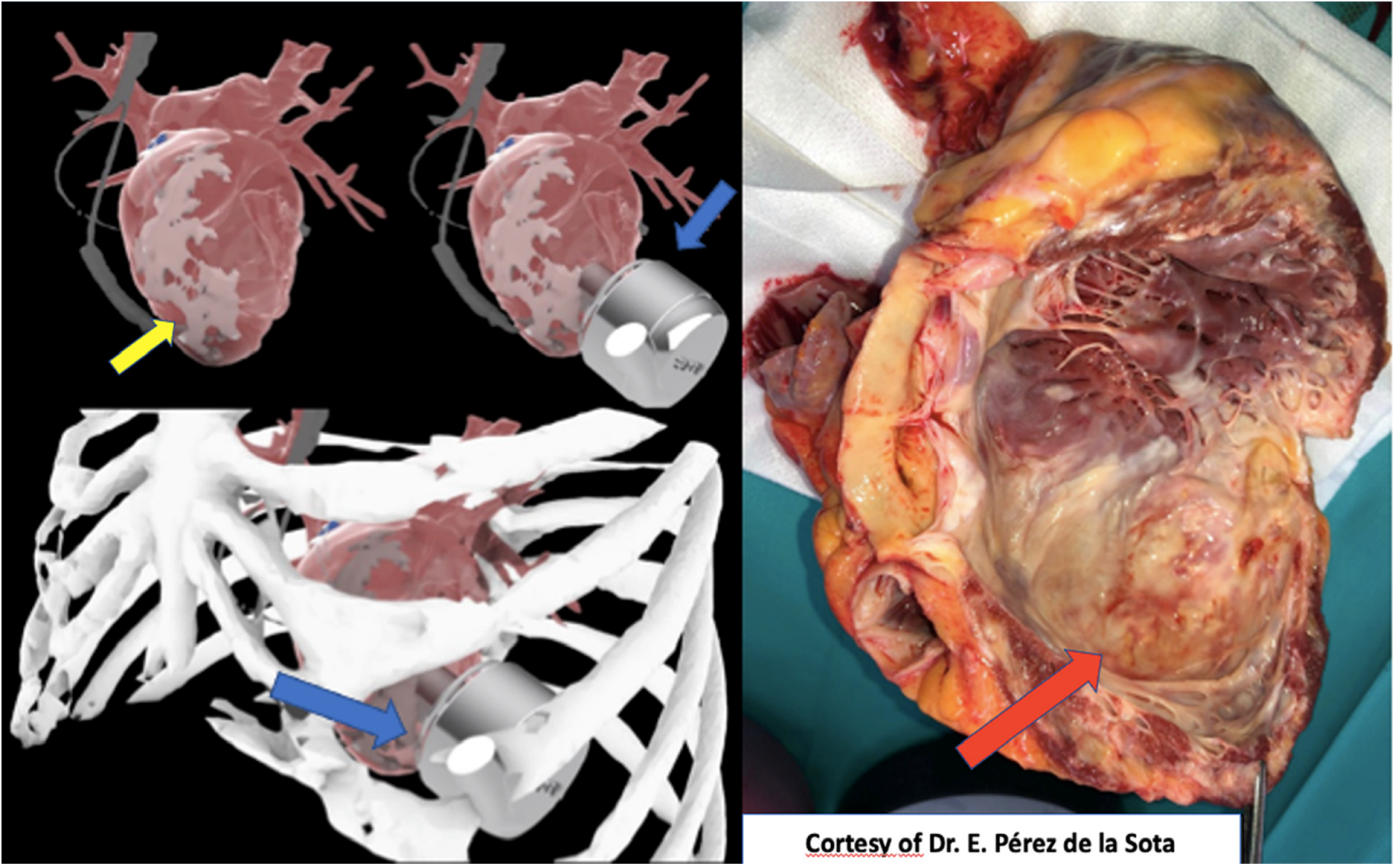
Left: 3D-reconstruction shows complex anatomy for left ventricular long term assistance device orientation. Yellow arrow shows the severe endocardial calcification at tipical inflow insertion place. Right: Explanted heart confirms severe endocardial calcification of apical and anterolateral wall of left ventricle. Red arrow shows the place of the aneurysm with severe calcification, confirming a great correlation with virtual model.

**Case 3**: Another case of LV aneurysm and extended endocardial calcification was analyzed by our Vall3DLab. After LV cavity reconstruction and virtual device implant, challenges to achieve optimal inflow drainage orientation were recognized. Even possible inflow cannula implantation at posterolateral wall was discussed, but finally patient was redirected to receive a heart transplantation, that was successfully performed some months later. Explanted heart confirmed challenges visualized by virtual implant: severe endocardial calcification of apical and anterolateral wall of LV, leaving posterior wall as possible inflow attachment only (Figure 6).

## Aortic root reconstruction

**Figure 7:**
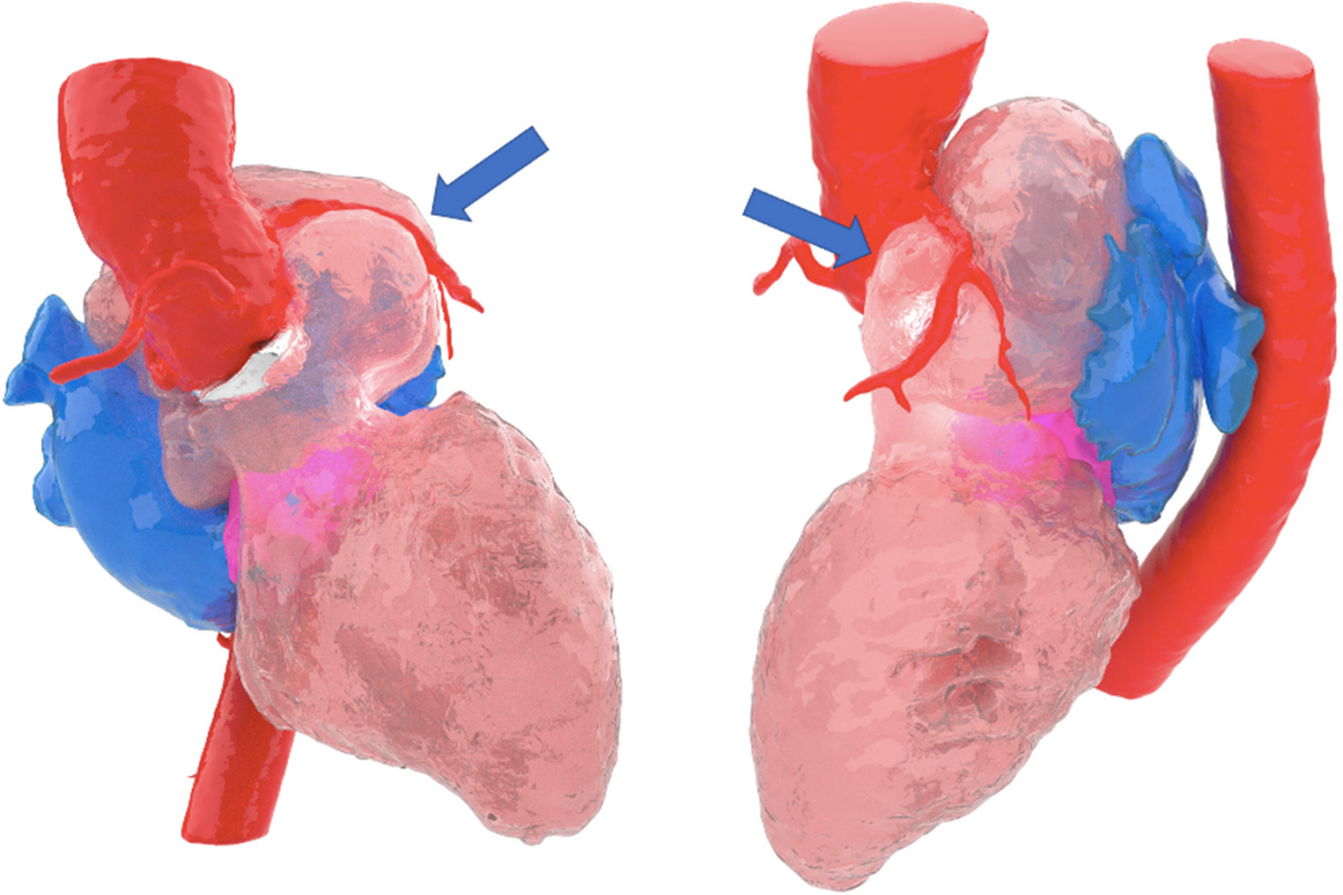
Blue arrow pointing image of large pseudoaneurysm, reconstructed by 3D modality.

**Case 4**: A 49-years-old female (170 cm, 95 kg,) with past clinical history of a staphylococcal infective endocarditis complicated with abscess in the aortic ring at the level of the left Valsalva sinus, with destruction of the left leaflet, undergoing valve replacement with a Carpentier Edwards Magna aortic bioprosthesis (21 mm) and closure of the abscessed cavity with a pericardial patch. Subsequent reoperation for annular abscess in the mitro-aortic junction, with replacement of the prosthesis with a St. Jude 25 mm mechanical valved tube (Bentall-DeBono procedure). During follow-up, 10 years after the intervention, development of a late complication of cardiac surgery with mitro-aortic dehiscence, severe mitral regurgitation, and formation of a large pseudoaneurysm just below the aortic prosthesis with displacement of the left atrium. Given the complexity of the planned treatment, virtual reconstruction and 3D biomodel had to be carried out to evaluate surgical challenges (see Figure 7). After reconstruction, taking all procedure related risks in account (expected long ischemia time, double valve replacement, aortic root replacement ± coronary surgery) and location of the false aneurysm, decision was made towards evaluation for heart transplantation as treatment option. Nowadays, patient is listed for heart transplantation in elective status.

## Results

In Table 1 we present the chosen treatment for each of the presented cases before and after AR/VR and 3D printing analysis. This shows how the usage of 3D biomodels has helped in finding and confirming viability of more suitable surgical approaches.

**Table 1:** Comparison of selected surgical approach before and after 3D biomodel analysis.

Case #	Case Description	Reconstruction chamber/point of interest	Intention to treat before virtual biomodel analysis (3D print, AR/VR)	Final treatment applied
1	Right Ventricular Cavity Reconstruction	Right ventricle chamber	Surgical Right ventricular assist device insertion via re-sternotomy approach	Use of percutaneous right ventricle assistance with clinical success
2	Left Ventricle Cavity Reconstruction	LV cavity/calcified LV aneurisms at theoretical implantable ventricular assist device inflow insertion point and orientation toward to mitral valve	Non-suitable for permanent implantable left ventricular assist device	Implant of HeartMate3 device using inflow insertion at point found during virtual reconstruction and performing surgical adaptation of position of device body at the chest, ongoing almost 4 years after intervention
3	Left Ventricle Cavity Reconstruction	LV cavity/calcified LV aneurisms at theoretical implantable ventricular assist device inflow insertion point and orientation toward to mitral valve	Non-suitable for surgical implantable left ventricular assist device, listed to heart transplantation	Finally Transplanted, but macroscopic evaluation of explanted heart confirmed possible implantation with inflow insertion at posterolateral LV wall originally planned in the 3D biomodel
4	Aortic Root Reconstruction	Aortic root aneurism	Hight risk complex conventional cardiac surgery	Listed for heart transplant

## Discussion

In recent years, there has been increasing interest in the use of 3D image reconstruction and virtual reality (VR) technology in the treatment of advanced heart failure. Our center has been using this technology since 2019 in order to plan complex heart anatomy treatment and perform mechanical circulatory device insertion or heart transplantation.

The use of 3D virtual models of the heart and heart assist devices, as well as the simulation of implant procedures using VR and 3D printed phantoms, has shown promise in improving the planning and execution of complex procedures. We have been using 3D reconstruction to create volumetric models of different heart chambers, such as the right ventricle, left ventricle, and aortic root, etc. These models are then used to virtually implant different types of circulatory assistance devices, which allows us to test the feasibility of the procedure, predict challenging procedure steps and even perform training. Additionally, these models can be used for both preoperative planning and surgical training. The use of 3D printed biomodels allows us to physically manipulate and test implant techniques. In this way, the use of VR reconstruction and 3D specific phantoms, as well as their combinate use, can help to identify potential problems and develop bail-out plans.

We have also been using extended virtual reality technology with the virtual glasses to create immersive, interactive 3D virtual models, which allow us to visualize the anatomy of the heart in a way that was previously not possible, and to plan and simulate implant procedures in a highly realistic manner. Our 3D laboratory expanded its activity through the last 3 years to the national level with high demand between heart failure physicians in the setting of this challenging advanced heart failure patients.

The results of our experience have been positive, with high estimation of success in selected adult patients with complex anatomy and need for short-term and long-term circulatory assistance device implantation, and cases with extremely high procedural risk.

## Future developments

The application of mixed reality to interventional medical procedures is promising. Its ability to interact with the surroundings allows this technology to locate medical images referenced to its actual anatomical true position. The interaction of mixed reality and ultrasound has been previously described (15). This ability might be of special interest to facilitate challenging invasive procedures as emergent Venoarterial Extracorporeal Membrane Oxygenation (vaECMO) cannulation: the mixed reality headset can overlay the ultrasound (US) image referenced to the US probe, so that the operator can easily locate the site of punction. Also, extended reality may provide a surgeon completely new and different ways to view complex pathology and prepare appropriate bail-out plans in setting of procedural complication. Several applications of mixed reality for cardiologic procedures are being developed or in the process of clinical adoption (16), and current evidence suggests that 3D-based planning (VR/AR, 3D-printing) results in improved clinical results (17).

## Conclusion

This work presents the important role of 3D printing, virtual intervention and use of extended reality for periprocedural planning in adults with advanced heart failure and complex anatomy requiring short-term or long-term mechanical circulatory support or heart transplantation. Although the presented imaging information is similar in traditional 2D screens, increased situational awareness and easier interaction with the models allow an easier planning of complex interventions. In the cases presented, the final approach decided for each patient after 3D-based planning was improved when compared to the intention to treat recorded before 3D-based planning.

## Limitations

Certain limitations should be considered. First of all, in the setting of acute heart failure and complex anatomy, a CT scan -or other imaging source data- is required for three-dimensional reconstruction and virtual model creation. Therefore, this technology is available in selected cases only, where clinical condition of the patient may give time for appropriate data imaging acquisition and analysis. In our center, the entire process between source image acquisition and delivery of printed 3D-model takes up to 48 h. Economic costs vary from model to model depending on the complexity, but in the reported cases have always stayed under 1.000 USD including materials, equipment usage and expert analysis time.

On the other hand, our laboratory database provided limited patient cohort. Therefore, we focused our work on the most representative advanced heart failure scenarios requiring three-dimensional and virtual models of VAD implantation. Nevertheless, our major finding of clinical utility of the described technology confirmed its important value even in the case cohort.

## Data Availability

The raw data supporting the conclusions of this article will be made available by the authors, without undue reservation.
